# Who Struck Billy Patterson?

**Published:** 1906-07

**Authors:** 


"WHO STRUCK BILLY PATTERSON?" "Donahue!" (Later: "I didn't say 'Donahue'; I said, 'I don't know who!'")

Dr. McCormack in his lecture said: "I found in my own home town (Louisville) six medical colleges where there should have been but one, schools whose faculties were fighting each other and whose students were trained in an atmosphere of slander and misrepresentation. No wonder a student entered his professional career with the impression that medical practice was a warfare, a life of strenuous self-assertion. He was usually confirmed in this opinion early in his practice by contact with the older members of the profession, men who would have been helpful, good men, but the young physician had no way of finding it out; whichever side of the older man he came upon proved to the wrong side for him."

.Believing that this quotation could not be correct, the editor of the *Louisville Medical Monthly* addressed Dr. McCormack the following letter:

LOUISVILLE, KY., April 20, 1906.

*Dr. J. N. McCormack, Bowling Green, Ky.*

DEAR DOCTOR: I have just had brought to my notice a clipping, of which the enclosed is a copy, purporting to come from the *Texas Courier-Record*, January, 1906.

I do not wish to do you the injustice to publish the enclosed without first ascertaining from you if you have been correctly quoted. If you have been correctly quoted, it is my intention to publish a general disclaimer of your first statement, and if you have not been correctly quoted, to publish the correct version of your speech. A prompt reply to this letter will be appreciated.

Very truly yours,

HENRY ENOS TULEY,
Editor.

REPLY:

CHICAGO, ILL., April 28, 1906.

DEAR DR. TULEY: Your note has just overtaken me here. The quotation is one of the stock phrases in my talk on organization and is fairly correct. It is part of my description of professional conditions in Kentucky *twenty-five years ago*, and then compared to similar conditions found in other cities and States, and with the improvement now found everywhere.

You may be sure that Kentucky does not suffer in the comparison, as I am a fairly loyal citizen. Run out to hear me at one of my appointments next month and hear for yourself. Cordially yours,
J. N. MCCORMACK.

[Was Dr. McCormack engaged in this work twenty-five years ago? This looks very much like crawfishing. — EDITOR "RED BACK."]

